# Efficacy of pegylated interferon α-2b plus entecavir therapy and predictors of treatment success in children with chronic hepatitis B

**DOI:** 10.3389/fimmu.2023.1282922

**Published:** 2023-12-04

**Authors:** Liang Huang, Hong Zhang, Xintong Kang, Zhu Chen, Lin Wang, Yilan Zeng

**Affiliations:** Department of Hepatology, Public Health Clinical Center of Chengdu, Chengdu, China

**Keywords:** pegylated interferon α-2b, entecavir, efficacy, chronic hepatitis B, predictors

## Abstract

**Introduction:**

Interferon therapy, used in the treatment of chronic hepatitis B (CHB), is one of the means by which patients can achieve a functional cure. Pegylated interferon is currently used in the treatment of CHB. There are two main types of pegylated interferon: α-2b and α-2a.

**Methods:**

This study explored the efficacy, safety, and predictors of treatment response for α-2b plus entecavir among children in a real-world setting.

**Results:**

The study included 76 patients aged 3–18 years, all of whom were treated with interferon α-2b plus entecavir. The mean duration of treatment was 401.99 days, and 31.6% (24/76) of patients achieved HBsAg clearance. Competing risk model analyses showed that children with baseline HBsAg <1500 IU/mL (subdistribution hazard ratio [sHR]=2.643, P=0.022) and a higher baseline alanine aminotransferase (ALT) level (sHR=1.005, P=0.000) had a higher probability of achieving HBsAg clearance during treatment. Conversely, children with a higher hepatitis B virus loading level (sHR=0.835, P=0.043) and age ≥10 years (sHR=0.243, P=0.002) had a lower probability of achieving HBsAg clearance during treatment. A decrease of >1 log_10_ in HBsAg level (sHR=3.479, P=0.001) at 12 weeks of treatment was associated with a higher probability of achieving surface antigen clearance.

**Discussion:**

These results indicated that interferon plus entecavir therapy is a promising means of achieving HBsAg clearance in children with CHB. Moreover, HBsAg, ALT, virus loading, and age are indicators of treatment success probability.

## Introduction

Interferons (IFNs) are immunomodulatory agents that can induce the expression of hundreds of genes, including genes with antiviral functions, thereby blocking viral replication at many levels (1). There is increasing interest in functional cures for hepatitis B virus (HBV) infection; treatments explored for this goal include IFNs, immune checkpoint inhibitors, therapeutic vaccines, and gene-engineered T cells (2). Notably, IFNs combined with nucleos(t)ide analogs (NUCs) or monotherapy have demonstrated promise in clinical practice, including a degree of efficacy in some adult patients and children with favorable characteristics (3).

There are two types of pegylated interferons (peg-IFNs): α-2a and α-2b. These types substantially differ in terms of chemical and structural characteristics, pharmacokinetic and pharmacodynamic properties, and dosing and administration (4). To our knowledge, there are limited available data regarding peg-IFN α-2b plus entecavir therapy for HBV infection in children. In this study, we analyzed 76 patients aged <18 years with CHB who received IFN α-2b plus entecavir to understand its efficacy and the predictors for treatment success.

## Materials and methods

### Patient enrollment

This study enrolled children with CHB who underwent treatment with IFN α-2b plus entecavir from 2021 to 2023. The inclusion criteria for this study were diagnosis with HBV infection between 3 and 18 years of age, no obvious signs of liver cirrhosis, and no other significant underlying diseases (e.g., psychiatric disorders, thyroid dysfunction, fundus abnormalities, or autoimmune diseases). The exclusion criteria were significant underlying diseases or contraindications to the use of IFN or entecavir.

### Study design

The treatment comprised peg-IFN α-2b plus entecavir: 180 µg peg-IFN α-2b/1.73 m^2^ per week and 0.0015 mg entecavir/kg per day. The peg-IFN α-2b was manufactured by Techpool Bio-pharma Co., Ltd., and the entecavir was manufactured by Chia Tai Tianging Pharmaceutical Group Co., Ltd. The study participants were evaluated approximately once per month to identify changes in medical indicators and the presence of any adverse reactions. After treatment completion, follow-up visits were initiated; participants continue to attend these visits. IFN strategies to achieve functional cures of HBV infection were optimized by response-guided treatment adjustment (RGT) rules (5, 6). These rules allowed doctors to decide whether to continue or discontinue treatment based on the patient’s hepatitis B surface antigen (HBsAg) response to the IFN treatment.

Three patients’ groups were defined in this study: the HBsAg NOT clearance group (group A), which was defined as patients who underwent treatment without HBsAg clearance; the HBsAg clearance group (group B), which was defined as patients who achieved HBsAg clearance; and the treatment discontinued group (group C), which was defined as patients whose treatment was discontinued by the doctors. The patients’ clinical data were organized in the same way as the survival data for analysis.

Ethical approval for this study protocol was obtained from the Ethics Committee of the Chengdu Public Health Clinical Center, and written informed consent was acquired from each patient before treatment. If the patients were over 8 years old, they needed to sign by themselves as well. The study complied with the ethical guidelines of the Declaration of Helsinki 2008.

### Follow-up

Baseline assessments included quantitative tests for HBsAg, hepatitis B surface antibody (HBsAb), hepatitis B e antigen (HBeAg), hepatitis B e antibody (HBeAb), and hepatitis B core antibody (HBcAb); blood routine, thyroid function, liver function, and renal function tests; and the detection of hepatitis B virus DNA (HBV-DNA). All patients underwent clinical follow-up and laboratory tests approximately once per month during the treatment process.

### Statistical analysis

A competing risk model was used to understand the impact of baseline status on HBsAg clearance. This type of model is useful in the context of censored data with competing risk. During treatment, doctors decide to discontinue treatment based on a patient’s response to IFN, typically when the patient shows a poor treatment response. In such instances, patients who discontinue treatment have a lower probability of achieving surface antigen clearance compared with patients who continue treatment at the same time point. Therefore, treatment discontinuation is considered a competing risk. The main endpoint in this study was the serological clearance of HBsAg, which is considered a “failure event” in the model. As noted above, treatment discontinuation was considered a “competing risk” in the model. The patients’ baseline features and early responses to treatment were entered into competing risk models. Multiple regression models were constructed using various combinations of independent variables. The best-fitting model was selected on the basis of statistical significance and professional expertise.

The Kaplan–Meier (KM) method and the log-rank test were used to compare the survival distributions of binary independent variables. Subdistribution hazard ratios (sHRs) of competing risk models were reported with 95% confidence intervals; all tests were two-sided, and the significant threshold was regarded as P < 0.05. All analyses were performed using Stata/MP 14.1 statistical software (StataCorp LLC, College Station, TX, USA).

## Results

### Baseline characteristics

This study included 76 patients [median age: 8 years (range, 3–18 years); 45 male and 31 female]. All included patients were Chinese citizens; 20 patients displayed Tibetan ethnicity, and the remaining 56 patients displayed Han ethnicity. Before treatment, the mean geometric value of HBV-DNA was 6.5 log_10_ IU/mL, the median HBsAg level was 12323 IU/mL, and the median alanine aminotransferase (ALT) level was 25 IU/L. According to our hospital’s standards (i.e., an upper limit of 37 IU/L), 59.2% (45/76) of patients had a normal ALT level. The neutrophil (NEU) and platelet (PLT) counts were all within normal ranges before treatment (**Table 1**). The univariate analysis of baseline characteristics indicated that the HBsAg and log HBV-DNA level were statistically different in three groups of patients. No other characteristics yielded positive results in the univariate analysis (**Table 1**).

**Table 1:** Baseline patient characteristics^1^.

Parameters	All patients	HBsAg NOT clearance group(A)N=20	HBsAg clearance group(B)N=24	Treatment discontinued group(C)N=32	P-values
Age(Years)	8^2^ (5-11)^3^	10.5^2^ (5-12.5)^3^	5.5^2^ (4.5-9)^3^	8.5^2^ (6-11.5)^3^	0.056^6^
Sex					
Male	45^4^ (59%^5^)	11^4^ (55%^5^)	12^4^ (50%^5^)	22^4^ (69%^5^)	0.338^6^
Female	31^4^ (41%^5^)	9^4^ (45%^5^)	12^4^ (50%^5^)	10^4^ (31%^5^)	
HBsAg(IU/mL)	24616^2^ (2607- 69434)^3^	19042^2^ (2481.15 -68958.98)^3^	2607^2^ (357.5-33641)^3^	42477.93^2^ (9467.5-72122.46)^3^	0.009^6^
HBeAg(IU/mL)	528.105^2^ (243.355-610.575)^3^	416.73^2^ (2.91-593.68)^3^	530.595^2^ (367.235-601.97)^3^	552.245^2^ (498.26-625.85)^3^	0.201^6^
ALT(IU/mL)	35^2^ (22.5- 54)^3^	30^2^ (23-49)^3^	45.5^2^ (26- 60)^3^	33^2^ (26-42.5)^3^	0.258^6^
WBC count(10^9^/L)	6^2^ (4.83-6.69)^3^	5.37^2^ (4.64-6.39)^3^	6.44^2^ (5.12-7.495)^3^	5.74^5^ (4.89-6.23)^3^	0.123^6^
PLT count(10^9^/L)	268.5^2^ (200.5-325)^3^	272^2^ (212-319.5)^3^	277^2^ (209.5-327)^3^	245^2^ (187.5-324.5)^3^	0.55^6^
NEU count(10^9^/L)	2.33^2^ (2.045-3.2)^3^	2.275^2^ (2.01- 3.055)^3^	2.9^2^ (2.13-3.485)^3^	2.315^2^ (2.065-3.125)^3^	0.375^6^
log_10_ HBV-DNA	6.5 (3-8)	6.5 (1.5-8)	4 (3-6)	7 (5.5-8)	0.005^7^
Tibetan ethnicity	20^4^ (26%^5^)	6^4^ (30%^5^)	5^4^ (21%^5^)	9^4^ (28%^5^)	0.801^7^
Previous antiviral treatment	5^4^ (7%^5^)	3^4^ (15%^5^)	2^4^ (8%^5^)	0^4^ (0%^5^)	0.057^7^
Immune-tolerant	67^4^ (88%^5^)	17^4^ (85%^5^)	21^4^ (88%^5^)	29^4^ (91%^5^)	0.903^7^

^1^(HBsAg), hepatitis B surface antigen; (HBeAg), hepatitis B e antigen; (SD), standard deviation; (IQR), interquartile range; (ALT), alanine aminotransferase; (WBC), white blood cell; (PLT), platelet. ^2^Median. ^3^IQR. ^4^Count data.^5^Rate. ^6^Kruskal–Wallis H test used. ^7^Fisher’s exact test used.

### Outcomes

**Figure 1:**
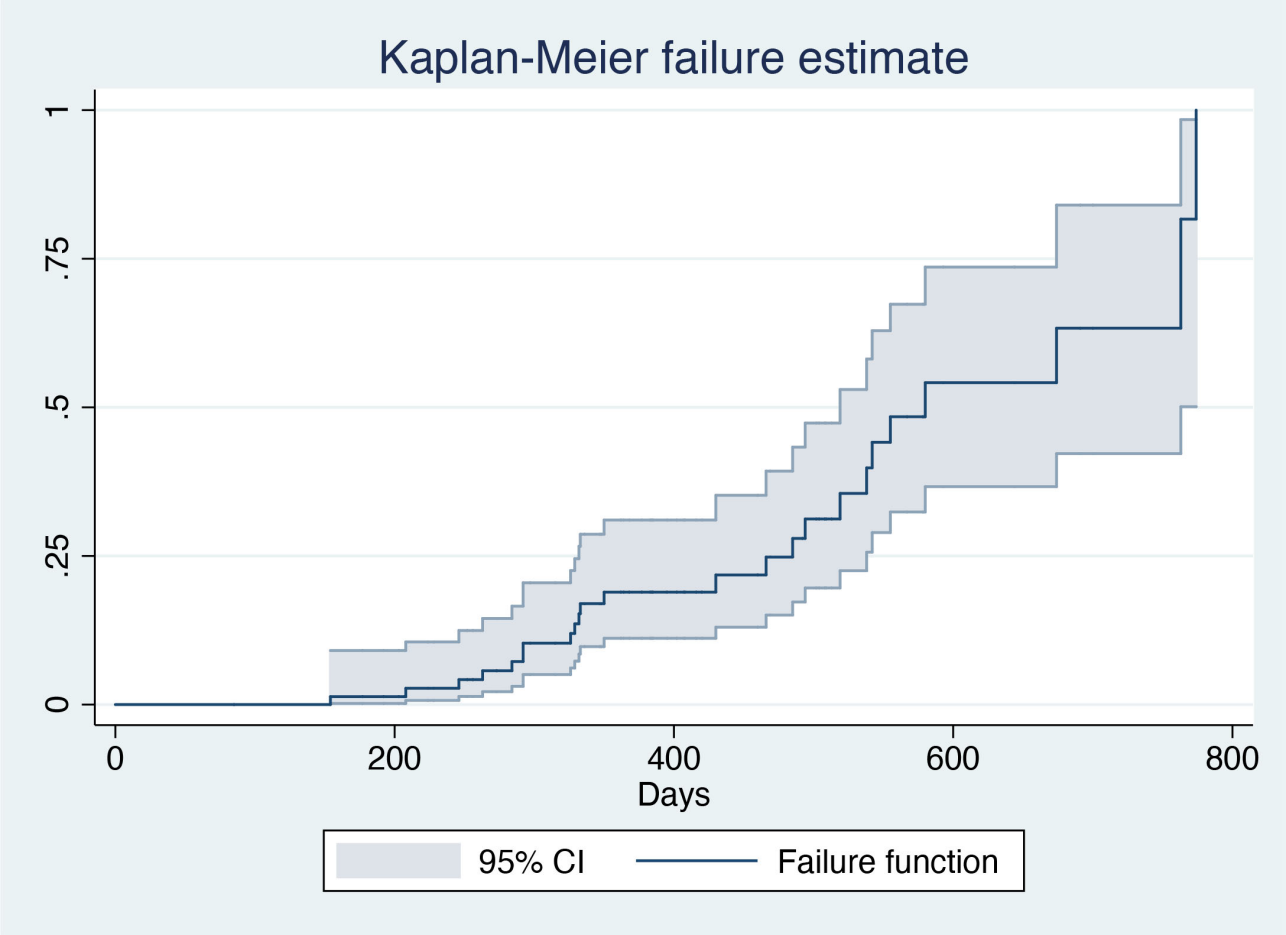
Kaplan–Meier failure estimates.

The mean duration of treatment was 401.99 days (standard deviation=147.08 days). Overall, 31.6% (24/76) of the patients achieved HBsAg clearance, with a median interval to HBsAg clearance of 245.5 days (interquartile range=149–354 days) (**Figure 1**; **Table 2**). In all, 26.3% (20/76) were in active treatment without HBsAg clearance, and 42.1% (32/76) discontinued the treatment due to doctors’ decisions. A total of 48 (63%) patients had a treatment duration longer than 48 weeks.

**Table 2:** Treatment outcomes^1^.

Parameters	0-24W	24-48W	48-72W	72-96W	>96W
Patients in the period	76	67	43	15	3
Cumulative HBsAg clearance	8	16	21	23	24
HBsAg clearance	8	8	5	2	1
Cumulative treatment discontinued	1	10	29	32	32
Cumulative rate of HBsAg clearance	10.5%	21.1%	27.6%	30.3%	31.6%
Rate of HBsAg clearance in the period	10.5%	12.0%	12.0%	13.3%	33.3%

^1^(W), weeks; (HBsAg), hepatitis B surface antigen.

### Competing risk model results

Independent variables were age ≥10 years or <10 years, baseline NEU and count, baseline ALT, baseline PLT count, surface antigen <1500 IU/mL or ≥1500 IU/mL, log_10_ HBV-DNA IU/mL at baseline, Tibetan ethnicity or non-Tibetan ethnicity, sex, immune tolerance status, and history of oral antiviral medication; these variables were included in the baseline competing risk model. Analysis of the model showed that the sHR for age ≥10 years was 0.243 (P=0.002), indicating that children aged ≥10 years are less likely to achieve surface antigen clearance. The sHR for surface antigen <1500 IU/mL was 2.643 (P=0.022), suggesting that patients with a surface antigen level <1500 IU/mL are more likely to achieve surface antigen clearance. The sHR for baseline viral load was 0.835 (P=0.043), indicating that patients with a higher baseline viral load are less likely to achieve surface antigen clearance. The sHR for baseline ALT levels was 1.005 (P=0.000), indicating that patients with a higher baseline ALT are more likely to achieve surface antigen clearance (**Table 3**).

An early response to treatment may also affect surface antigen seroconversion. Therefore, we constructed a model that considered whether the surface antigen level decreased by 1 log_10_ at 12 weeks of treatment; the rates of decrease in white blood cell count, NEU count, and PLT count; and the rate of ALT elevation. Through variable selection in the model, we found that a single log_10_ decrease in surface antigen at week 12 was associated with surface antigen clearance during treatment (**Table 3**). Other early response factors analyzed did not display statistical significance and were excluded from the model. The calculation of the rate of change was rate = (value after first month – baseline)/baseline value.

**Table 3:** Competing risk model results^1^.

Variables	sHR	SE	Z	P-values	95% CI	
Baseline model						
≥10 Years old	0.243	0.109	-3.150	0.002	0.101	0.586
HBsAg<1500IU/mL	2.643	1.119	2.290	0.022	1.152	6.062
Baseline Virus loading	0.835	0.074	-2.030	0.043	0.701	0.994
Baseline ALT	1.005	0.001	3.820	0.000	1.002	1.008
Log pseudo-likelihood = -78.14, P=0.00						
Early response model						
12-weeks HBsAg decrease >1 log_10_	3.479	1.357	3.200	0.001	1.620	7.471
Log pseudo-likelihood = -87.10, P=0.00						

^1^(sHR), subdistribution hazard ratio; (SE), standard error; (CI), confidential interval.

### Kaplan–Meier analysis

**Figure 2:**
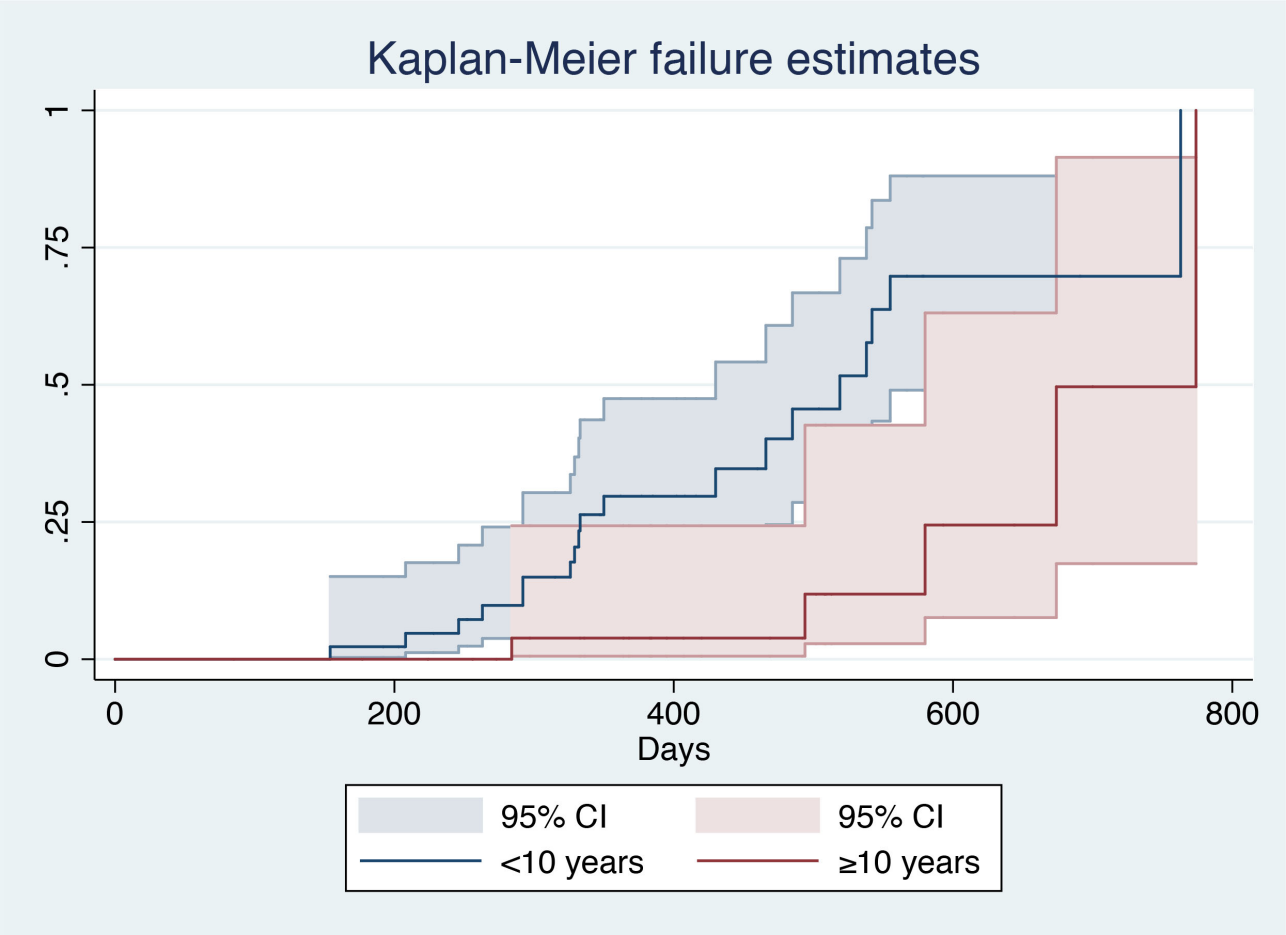
Kaplan–Meier analysis of age^1^. ^1^(CI), Confidential interval.

After patient stratification according to age (≥10 years vs. <10 years), log-rank analysis showed that patients aged <10 years had a larger area under the curve (AUC) (χ^2^ = 9.36, P=0.0022), indicating a higher probability of HBsAg clearance in such patients (**Figure 2**).

**Figure 3:**
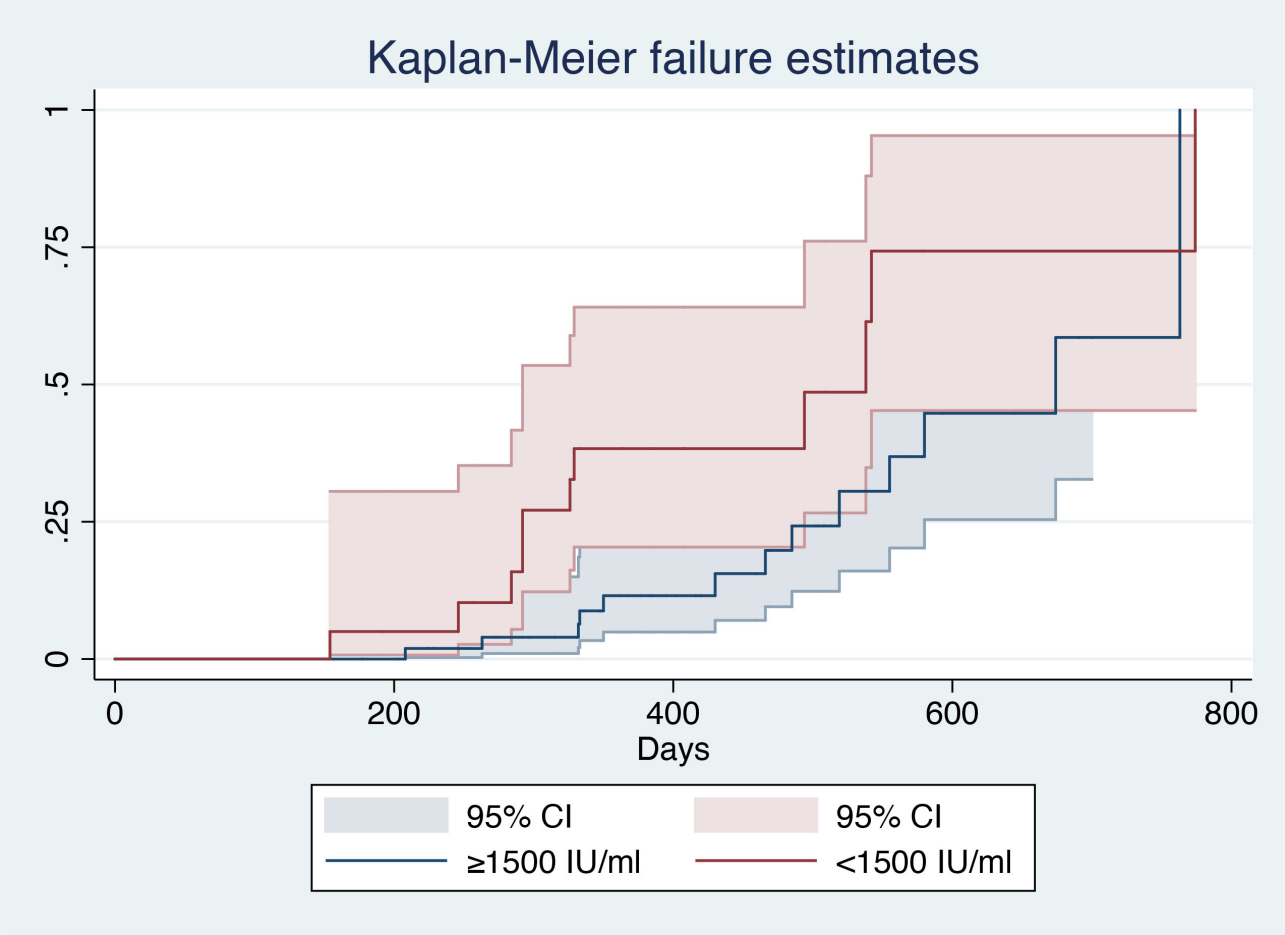
Kaplan–Meier analysis of baseline HBsAg level^1^. ^1^(CI), Confidential interval; (HBsAg), hepatitis B surface antigen.

After patient stratification according to baseline surface antigen level (<1500 IU/mL vs. ≥1500 IU/ml), log-rank analysis showed that patients with a baseline HBsAg level ≥1500 IU/mL had a smaller AUC (χ^2^ = 4.7, P=0.0302), indicating a lower probability of HBsAg clearance in such patients (**Figure 3**).

**Figure 4:**
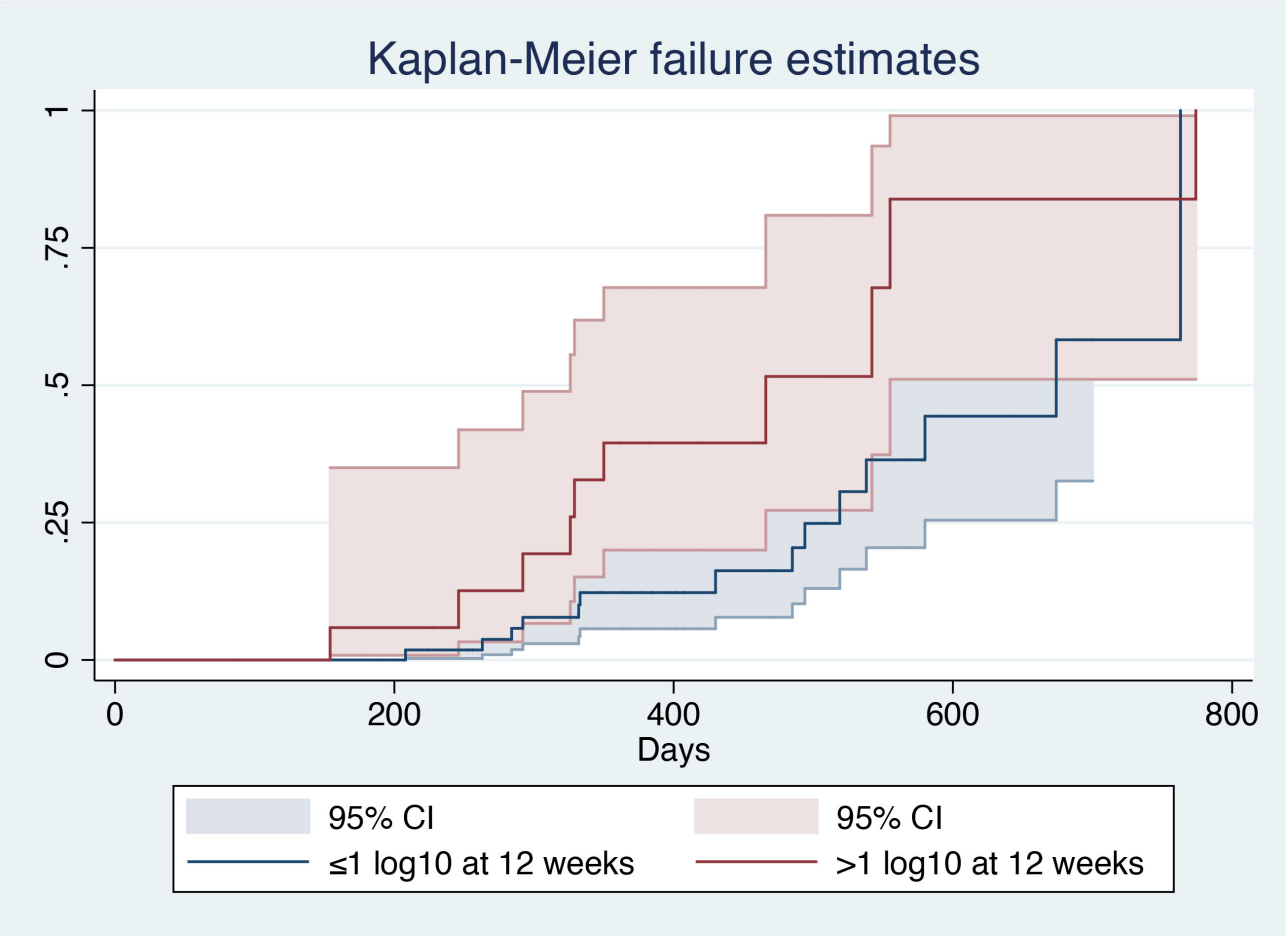
Kaplan–Meier analysis of decrease in baseline HBsAg level^1^. ^1^ (CI), Confidential interval.

After patient stratification according to the decrease in baseline HBsAg level at 12 weeks (>1 log_10_ vs ≤1 log_10_), log-rank analysis showed that patients with a 1 log_10_ decrease in baseline HBsAg level had a larger AUC (χ^2^ = 4.57, P=0.0326), indicating a higher probability of HBsAg clearance in such patients (**Figure 4**).

### Follow-up results among patients who achieved HBsAg clearance

Among the 24 patients who achieved surface antigen clearance, 11 completed follow-up HBsAb tests after treatment finished, indicating that they had at least one systematic evaluation of HBV infection within 3 months after treatment finished. Among the patients who achieved HBsAg clearance, 75% had a positive HBsAb test result (≥10 mIU/mL). In these patients, the median surface antibody level was initially 51.13 mIU/mL. At a mean interval of 75.83 days after treatment, the median surface antibody level increased to 304 mIU/mL (upper limit of detection: 1000 mIU/mL).

### Major safety concerns

Among all 76 patients, three (4%) experienced thyroid dysfunction during the treatment process, leading to temporary treatment discontinuation, but they successfully recovered and continued treatments. Of the patients, 78% (59/76) had an elevated ALT level, 76% (58/76) had a decreased NEU count, and 71% (54/76) had a decreased PLT count. Statistical analyses using paired rank-sum tests were conducted to compare key safety parameters (e.g., ALT, NEU count, and PLT count) at baseline, after 1 month of treatment, last observation during treatment (mean time: 357.99 days), and follow-up after treatment completion (mean time: 80.44 days after treatment completion, n=29 patients). When ALT levels after 1 month of treatment and at the last observation during treatment were compared with baseline, we observed a significant increase in ALT. However, during the follow-up period, ALT levels returned to baseline. In contrast, both NEU and PLT counts decreased after 1 month of treatment and at the last observation during treatment; during the follow-up period, both counts returned to baseline levels (**Table 4**). Other minor adverse events included fever, poor appetite, skin rash, and fatigue.

**Table 4:** Analysis of major safety parameters of blood routine^1^.

Parameters	Baseline	One month	Last observation	Follow-up
ALT (IU/mL)	35^2^ (24-54)^3^	64.5^2^ (38-128) ^3^ 0.00^4^	62^2^ (35-99) ^3^ 0.02 ^4^	25^2^ (16-42) ^3^ 0.43 ^4^
NEU (10^9^/L)	2.33^2^ (2.05-3.2) ^3^	1.81^2^ (1.35-2.52) ^3^ 0.00 ^4^	1.81^2^ (1.45-2.54) ^3^ 0.00 ^4^	3.09^2^ (2.01-3.97) ^3^ 0.88 ^4^
PLT (10^9^/L)	268.5^2^ (200.5-325) ^3^	195^2^ (141.5-273) ^3^ 0.00 ^4^	165^2^ (123- 217) ^3^ 0.00 ^4^	231^2^ (164-312) ^3^ 0.88 ^4^

^1^(WBC), white blood cell; (PLT), platelet; (NEU), neutrophil.

^2^ medians, ^3^ interquartile range. ^4^P-value vs. baseline.

## Discussion

### Efficacy and predictors of treatment success

In this study, competing risk model analyses suggested that a baseline HBsAg level <1500 IU/mL and a higher baseline ALT level were associated with a higher probability of surface antigen clearance during treatment (sHR=2.643, P=0.022). Additionally, age ≥10 years and a higher baseline virus loading (HBV-DNA) level were associated with a lower probability of HBsAg clearance (sHR=0.243, P=0.002 and sHR=0.835, P=0.043) during treatment. A baseline HBsAg level ≤1500 IU/mL and a higher ALT level associated with a higher HBsAg clearance rate during treatment. A decrease of >1 log_10_ in HBsAg level (sHR=3.479, P=0.001) at 12 weeks of treatment was associated with a higher probability of achieving surface antigen clearance. The results indicated that the baseline HBsAg level and virus loading were clinically important factors. The younger patients had a greater chance of achieving HBsAg clearance during treatment. The early response of HBsAg during treatment was a good predictor for the treatment success. However, the immune tolerance status was not statistically significant. That means immune tolerance may not closely correlated to the HBsAg clearance rate in younger patients.

In all, 31.6% (24/76) of the patients achieved HBsAg clearance. The results indicated the interferon α-2b plus entecavir therapy is a promising means for HBsAg clearance in children with CHB.

### Literature reviews

Entecavir, approved for the treatment of children with hepatitis B, has a strong inhibitory effect on HBV-DNA (7) but a relatively weak effect on HBsAg clearance. In contrast, IFN is an important conventional antiviral agent for the treatment of hepatitis B, which achieves its effects through immunomodulation and the reduction of HBsAg expression. Combination therapy involving these two drugs can increase the therapeutic effect and lead to a functional cure in some patients. Notably, one study showed that IFN therapy can reduce the overall risk of hepatocellular carcinoma (8). In the present study, we found that the combination of peg-IFN α-2b and entecavir yielded relatively good efficacy and safety. Among patients who received treatment over a mean duration of 401.99 days, the HBsAg clearance rate was 32%.

Various studies have reported dissimilar outcomes regarding the efficacy of IFN in the treatment of hepatitis B (**Table 5**). Among children in the immune-active phase, studies using IFN monotherapy have achieved surface antigen seroconversion rates of 11.1%–22.2% at the end of treatment (10, 14). Among children in the immune-tolerant phase, a study using IFN α-2a plus entecavir indicated a very low rate of surface antigen clearance and a high incidence of adverse reactions (12). However, another study (conducted in China) showed that combination therapy comprising lamivudine and IFN could achieve better results among children in the immune-tolerant phase, with a surface antigen clearance rate of 21.74% at the end of treatment (11).

**Table 5:** Literature reviews regarding the efficacy of IFN treatment for hepatitis B for children^1^.

Author	time	Treatment	Duration	Patient	N	Results
Sokal (9)	1998	IFN α-2b	48W	children	144	HBeAg clearance:26%
Hu (10)	2019	IFN α-2b vs peg-IFN α-2a	48W	children	36	HBsAg clearance: 22.2% vs. 11.1%
Zhu (11)	2018	IFN+LAM	96W	Immune tolerant	69	HBeAg seroconversion: 32.61% HBsAg clearance: 21.74%
Bortolottia (3)	2000	IFN α-2a	3-6 months	children	107	HBsAg clearance:25% for responders (16 patients)
Fan (12)	2019	peg-IFN α-2a	48W	children	41	HBeAg clearance: 66.7% HBsAg clearance: 52%
Rosenthal (13)	2019	peg-IFN α-2a +ETV	48W	Immune tolerant	60	HBsAg clearance:3%
Wang (14)	2021	IFN α	72W	Immune active	411	HBeAg clearance:39.87% HBsAg clearance:11.08%
Choe (15)	2007	IFN α+ LAM	12 months	children	59	HBeAg clearance:65%
D’Antiga (16)	2006	IFN α+LAM	10 months	Immune tolerant	23	HBsAg seroconversion:17%
Paddar (17)	2012	IFN α+LAM	52 weeks	Immune tolerant	28	HBsAg seroconversion:21.4%

^1^(peg-IFN), pegylated interferon; (LAM), lamivudine; (HBsAg), hepatitis B surface antigen; (HBeAg), hepatitis B e antigen.

Three studies utilizing peg-IFN (peg-IFN α-2b monotherapy in one and peg-IFN α-2a in the others) yielded surface antigen clearance rates ranging from 5% to 52%. Notably, Fan et al. reported an HBsAg clearance rate of 52%. In that study, the patients exhibited a high mean baseline ALT level (126 IU/mL) (12). These findings are consistent with our results, which indicated that the probability of achieving HBsAg clearance increased with increasing baseline ALT level. Notably, all previous studies used interventions that considerably differed from the pegylated IFN α-2b plus entecavir strategy used in the present study.

### Immunology status

There remains debate regarding whether antiviral therapy is necessary for children with CHB in the immune-tolerant phase. The European Association for the Study of the Liver (EASL) did not suggest antiviral treatment or liver biopsy for such children (18). The European Society for Pediatric Gastroenterology Hepatology and Nutrition only recommends performing a liver biopsy if the ALT level is persistently elevated; it recommends only providing treatment to children with at least moderate inflammation and fibrosis or children with a positive family history of hepatocellular carcinoma (19). Patients in the immune-tolerant phase of disease may exhibit minimal fibrosis and inflammation, and they may display limited immune efficacy against HBV compared with other patients (20). However, a considerable proportion of patients in the immune-tolerant phase display progressive liver fibrosis and inflammation (21, 22); persistently high levels of HBV-DNA over many years likely cause a gradual increase in the risk of hepatocellular carcinoma, despite the absence of active liver inflammation and fibrosis (22). Guidelines from the EASL recommend treatment for patients with HBeAg-positive CHB, defined by persistently normal ALT and high HBV-DNA levels, if those patients are aged ≥30 years; the histological severity of liver lesions does not change this recommendation. The EASL guidelines also indicate that the course of disease is generally mild, and most affected children do not meet the standard indications for treatment (18). The American Association for the Study of Liver Diseases (AASLD) supports the avoidance of treatment for patients (including children) in the immune-tolerant phase (23). Thus, there is some evidence that treatment for children in the immune-tolerant phase is generally not supported; however, a treatment trial achieved limited effects in these children (12). However, there is new evidence of underlying active disease, based on pathological and virological analyses (21). A high frequency of HBV-DNA integration was identified by inverse polymerase chain reaction. Random insertions of HBV-DNA result in genomic instability and have been associated with hepatocarcinogenesis (24).

Concerning children in the immune-tolerant phase, there have been some reports concerning the use of IFN therapy of children, but response rates considerably vary. In 2006, one study showed that treating children in the immune-tolerant phase with IFN plus lamivudine resulted in an HBeAg seroconversion rate of 22% and an HBsAg seroconversion rate of 17% (16). In 2012, a study showed that treatment with lamivudine plus IFN resulted in an HBsAg clearance rate of 21.4% (17). However, a 2019 study showed that an HBsAg clearance rate of only 3% was achieved with entecavir plus IFN (12). In the present study, most patients (88%) were in the immune-tolerant phase. Our results showed no significant differences in HBsAg clearance between patients in the immune-tolerant phase and those who were not (31% vs 33%, P=1.00 according to Fisher’s exact test). Furthermore, competing risk model analysis showed that the phase of disease (immune-tolerant or not) was not statistically significant. The results suggest the need for a rethink of the immune-tolerant phase in children.

### Safety concerns

Notably, three (4%) patients in the present study experienced thyroid dysfunction during treatment. Patients with an elevated ALT level and decreased blood cell counts were able to recover after treatment discontinuation. Overall, adverse reactions to IFN therapy require close monitoring. Compared with oral antiviral drugs, IFN therapy involves many adverse reactions, such as an elevated ALT level, decreased blood cell counts, fever, skin allergies, fever, and fatigue; however, these adverse reactions gradually improve or completely resolve after IFN discontinuation. Although most adverse reactions to IFN therapy are reversible, the use of IFN strategies in children should be carefully considered before implementation (25, 26).

## Data availability statement

The data supporting the findings of this study are available from the corresponding author YZ on request.

## Ethics statement

The studies involving humans were approved by the Ethics Committee of the Chengdu Public Health Clinical Center (reference number: [2018Y]001), and complied with the ethical guidelines of the Declaration of Helsinki 2008. The studies were conducted in accordance with the local legislation and institutional requirements. Written informed consent for participation in this study was provided by the patients’ parents and by the patients who were over 8 years old.

## Author contributions

LH: Conceptualization, Data curation, Methodology, Writing – original draft. HZ: Conceptualization, Data curation, Investigation, Writing – review & editing. XK: Conceptualization, Data curation, Writing – review & editing. ZC: Conceptualization, Data curation, Writing – review & editing. LW: Conceptualization, Investigation, Writing – review & editing. YZ: Conceptualization, Writing – review & editing.
